# Using safe, affordable and accessible non‐steroidal anti‐inflammatory drugs to reduce the number of HIV target cells in the blood and at the female genital tract

**DOI:** 10.1002/jia2.25150

**Published:** 2018-07-26

**Authors:** Julie Lajoie, Kenzie Birse, Lucy Mwangi, Yufei Chen, Juliana Cheruiyot, Maureen Akolo, John Mungai, Genevieve Boily‐Larouche, Laura Romas, Sarah Mutch, Makobu Kimani, Julius Oyugi, Emmanuel A Ho, Adam Burgener, Joshua Kimani, Keith R Fowke

**Affiliations:** ^1^ Department of Medical Microbiology and Infectious Diseases University of Manitoba Winnipeg MB Canada; ^2^ Department Medical Microbiology University of Nairobi Nairobi Kenya; ^3^ National HIV and Retrovirology Labs JC Wilt Center for Infectious Diseases Public Health Agency of Canada Winnipeg MB Canada; ^4^ College of Pharmacy University of Manitoba Winnipeg MB Canada; ^5^ Partners for Health and Development in Africa Nairobi Kenya; ^6^ School of Pharmacy, University of Waterloo Waterloo ON Canada; ^7^ Unit of Infectious Diseases Department of Medicine Solna Center for Molecular Medicine Karolinska Institute Karolinska University Hospital Stockholm Sweden; ^8^ Department of Community Health Science University of Manitoba Winnipeg MB Canada

**Keywords:** HIV prevention, acetylsalicylic acid, hydroxychloroquine, immune quiescence, immune activation, HIV‐exposed seronegative (HESN), inflammation, HIV target cells

## Abstract

**Introduction:**

At its basic level, HIV infection requires a replication‐competent virus and a susceptible target cell. Elevated levels of vaginal inflammation has been associated with the increased risk of HIV infection as it brings highly activated HIV target cells (CCR5+CD4+ T cells; CCR5+CD4+CD161+ Th17 T cells) to the female genital tract (FGT) where they interact with HIV. Decreased HIV risk has been associated with a phenotype of decreased immune activation, called immune quiescence, described among Kenyan female sex workers who were intensely exposed to HIV yet remain uninfected. Current prevention approaches focus on limiting viral access. We took the novel HIV prevention approach of trying to limit the number of HIV target cells in the genital tract by reducing inflammation using safe, affordable and globally accessible anti‐inflammatory drugs.

**Methods:**

We hypothesized that the daily administration of low doses of acetylsalicylic acid (ASA 81 mg) or hydroxychloroquine (HCQ 200 mg) would reduce inflammation thereby decreasing HIV target cells at the FGT. Low‐risk HIV seronegative women from Nairobi, Kenya were randomized for six weeks therapy of ASA (n = 37) or HCQ (n = 39) and tested to determine the impact on their systemic and mucosal immune environment.

**Results:**

The results showed that HCQ use was associated with a significant reduction in the proportion of systemic T cells that were CCR5+CD4+ (*p *= 0.01) and Th17 (*p* = 0.01). In the ASA arm, there was a 35% and 28% decrease in the proportion of genital T cells that were CD4+CCR5+ (*p* = 0.017) and Th17 (*p* = 0.04) respectively. Proteomic analyses of the cervical lavage showed ASA use was associated with significantly reduced amount of proteins involved in the inflammatory response and cell recruitment at the mucosa, although none of the individual proteins passed multiple comparison correction. These changes were more apparent in women with *Lactobacillus* dominant microbiomes.

**Conclusion:**

Together, these data indicate that taking low‐dose ASA daily was associated with significant reduction in HIV target cells at the FGT. This study provides proof‐of‐concept for a novel HIV‐prevention approach that reducing inflammation using safe, affordable and globally accessible non‐steroidal anti‐inflammatory agents is associated with significant reduction in the proportion of HIV‐target cells at the FGT.

## 1 Introduction

Despite efforts to increase the access to prevention programmes, there are over 1.5 million new HIV infections annually 1. In Africa, it is estimated that women have a twofold greater risk for HIV acquisition during unprotected vaginal intercourse compared with men 1, and female sex workers (FSW) are 13.5 times more likely to become HIV infected compared to other women 2. Therefore, developing new HIV prevention methods that would empower women to protect themselves is a priority for public health.

One of the biological factors most strongly associated with increased HIV risk is the presence of inflammation prior to HIV exposure. The presence of sexually transmitted infections (STIs) and diverse bacterial communities during bacterial vaginosis are associated with increased risk of HIV infection 3. Diverse vaginal bacterial communities are thought to increase HIV risk through mechanisms including immune activation (IA), recruitment of HIV target cells 4, as well as damage to epithelial barriers 5. The CAPRISA 004 study showed that women with greater IA were at higher risk of HIV acquisition regardless of study arm 6, which is likely linked to the presence of dysbiotic bacteria that metabolize tenofovir, rendering it ineffective 7.

Our group showed that decreased risk of HIV infection among the Pumwani Sex Worker Cohort from Kenya was associated with an immune phenotype called immune quiescence (IQ) 8. This phenotype is characterized by lower baseline level of T cell IA 9 and pro‐inflammatory chemokines 10 which decreases the number of HIV target cells (CCR5+ CD4+ T cells) at the genital tract 11.

With the goal of reducing HIV‐target cells at the genital tract, we sought to reduce inflammation and induce the IQ phenotype using a pharmacological approach. The criteria for the anti‐inflammatory drugs chosen were that they had to be FDA‐approved with a long safety track record, affordable and widely accessible in all parts of the world. We conducted a pilot study in Kenya with the objectives of assessing if the non‐steroidal anti‐inflammatory drugs acetylsalicylic acid (ASA) or hydroxychloroquine (HCQ) could reduce T cell IA, HIV target cell numbers and inflammation at the genital tract.

## 2 Methods

### 2.1 Study design and participants

**Figure 1:**
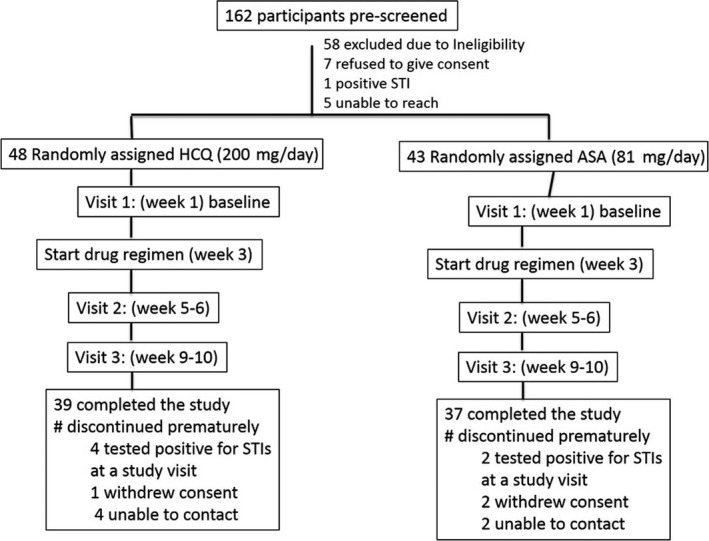
Schematic of the trial profile. HCQ, hydroxychloroquine; ASA, acetylsalicylic acid.

Inducing IQ (IIQ) study was a randomized pilot, open‐label study conducted at the Pumwani Maternity and Baba Dogo community clinics located in Nairobi, Kenya. We enrolled 91 HIV uninfected women who were from the general community, that is not commercial sex workers, with 39 and 37 completing the study in the HCQ and ASA arms respectively (Figure 1). Both the University of Nairobi and the University of Manitoba ethical review boards approved this study and participants provided written informed consent.

### 2.2 STI screening

At the screening visit and each study visit, vulvovaginal swab was collected to determine the presence of candida pseudohyphae, bacterial vaginosis and Trichomonas vaginalis. Urine samples were collected for PCR detection of *Neisseria gonorrhoea* and *Chlamydia trachomatis* (Roche Amplicor kits, Indianapolis, IN, USA). Syphilis testing was performed at screening visit, all women were tested negative. HIV serology using a rapid test (Determine, Inverness Medical, Japan) was performed at the first and last study visit.

### 2.3 Study procedures

Participants were randomized to receive either oral ASA (81 mg) (Bayer Canada, Mississauga, ON, Canada) or HCQ (200 mg) (Sanofi Aventis, Paris, France) once a day for a period of six weeks. Participants were pre‐screened for STIs including HIV. Participants were followed up for a total of 12 weeks (four pre‐drug, six on drug, two post‐drug) with sampling occurring monthly. Women not on depot medroxyprogesterone acetate (DMPA) were asked to come 5 to 8 days after the end of menstrual bleeding to ensure that all mucosal samples were taken during the follicular phase of the cycle. Visit 1 was the pre‐drug visit and visit 3 was following six weeks on drug therapy.

### 2.4 Adverse events

Of the participants randomized to the ASA arm (n = 37), the drug was well tolerated at the week two visit with 35 participants (92%) having no adverse events. Three reported minor adverse events (one experienced headache and abdominal discomfort in the first four days of taking the drug; one reported epigastric pain, abdominal fullness and heartburn. While the third did not specify what type of adverse event they had). After six weeks of taking ASA, the majority of participants 33 (86%) did not report an adverse event. Five participants reported an effect (two indicated nausea, one of joint pains for five days, one of coughing while one did not specify). Only one participant indicated an effect at both follow‐up visits.

Among the HCQ‐randomized participants (n = 38), 32 (84%) did not experience any side effect. Six reported experiencing a side effect at the two‐week follow‐up visit. The side effects reported at visit 2 were headache (four participants), nausea (one participant), dizziness and stomach ache (one participant). After six weeks of HCQ, 26 (68%) of the participants indicated they did not experience any adverse event. Twelve participants reported mild adverse events including abdominal pain (three participants), dizziness (two participants), throat pain (one participant), skin rash (two participants), loss of appetite (one participant) and boils (one participant). Three participants indicated having a side effect at both visit 2 and visit 3 follow ups. One participant indicated that she did not take the drug daily as per the study protocol because of throat pain.

### 2.5 Sample collection and processing

Blood, cervicovaginal lavage (CVL) and cervical mononuclear cells (CMC), collected by cytobrush, were obtained from all participants at visit 1 (baseline) and visit 3. CVL was collected first followed by cervical cytobrushes as in Juno et al. 12. Peripheral blood mononuclear cells (PBMC) were isolated by Ficoll density gradient.

### 2.6 PBMC and CMC flow cytometry

PBMC (10^6^) and CMC were washed with 2% FBS‐1× PBS and stained with PE.Cy5‐CD3, FITC‐CD4, PE‐CD95, APC.H7‐HLA‐DR, APC‐CD161, V450‐CCR5, PE.Cy7‐CD69 (BD Biosciences, San Jose, CA, USA), and Far Red‐Live Dead discriminant (Invitrogen, Carlsbad, CA, USA). Data were acquired on an LSRII flow cytometer (BD BioSciences) and analysed using FlowJo v10.0.8r1 (TreeStar, Ashland, OR, USA). Cytokines and chemokines in the blood were detected by Millipore micro‐bead array assay for the following cytokines (IFN‐γ, IL‐10, IL‐12p70, IL‐15, sCD40L, IL‐17A, IL‐1α, IL‐1β, IL‐2, IL‐8, IP‐10, MCP‐1, MIP‐1α, MIP‐1β, TFN‐α, MIG, IL‐1Rα, MIP‐3a, IL‐2RA.)

### 2.7 Proteome and microbiome analysis

Mass spectrometry was utilized to characterize both human and microbial proteins as described previously 7, 13. Briefly, 100 μg of protein from each sample were digested with trypsin and analysed by tandem mass spectrometry using an Orbitrap Velos mass spectrometer (ThermoFisher Scientific, Waltman, MA, USA). Human peptide identity searching was performed using Mascot Daemon v2.4.0 (Matrix Science Inc., Boston, MA, USA) against the SwissProt database restricting taxonomy to Human. Bacterial peptide identity searches were performed using a manually curated TrEMBL database containing the major identified genera identified from an initial search (17 genera total, 4,206,764 proteins total) (see Appendix S1 for additional details).

### 2.8 Statistical analysis

The analyses performed for this pilot study included assessment of the difference in T cell IA between baseline and visit 3. The primary outcome was the assessment of HIV target cells, defined as CD4+CCR5+CD3+ and CD4+CD161+CCR5+, and their activation phenotype (CD69+). The study was not designed to compare between the two drug arms. In the analysis, the χ^2^ test was used to assess the significance of the associations between categorical variable using Prism 6.0f (GraphPad Software, La Jolla, CA, USA) and Gaussian distribution was tested by Shapiro‐Wilk normality test and normality plot using SPSS (IBM, Armonk, NY, USA). See Appendix S1 for details on which factor were normally distributed. To compare baseline to visit 3, two‐tailed paired T test or Wilcoxon matched‐pair signed‐rank test were performed. Using SSPS, multivariate linear regressions were built to determine if hormonal contraception and age impacted the change score of the intercept (visit 3‐baseline). Pearson correlation or Spearman's rank test were used for correlations between continuous variables. Generalized linear models were performed with the stats package in R (version 3.3.1) to determine interactions effects between study treatment and microbiome state on protein expression (R Foundation for Statistical Computing, Viennna, Austria). As this was a discovery study, *p* values were considered significant if ≤0.05. The study was monitored by a Data and Safety Monitoring Board derived from University of Manitoba experts. The study is registered on ClinicalTrials.gov (NCT02079077). Participants who were discontinued from the study were excluded from the analysis.

## 3 Results

### 3.1 Participant characteristics

A total of 91 women (Figure 1) were enrolled in the study and followed up between May and December 2014. In total, 76 women completed all the study visits; (39 in the HCQ arm and 37 in the ASA arm). All the women remained HIV seronegative at follow‐up testing at the end of the study. Participant characteristics at entry were similar between the study arms (Table 1).

**Table 1:** Sociodemographic factors

	ASA group n = 38	HCQ group n = 39
Age (mean ± SD)	32 ± 8	30 ± 6
Practicing vaginal douching	21	20
Hormonal contraception		
No HC	12	16
Progesterone‐based	21	19
Oral pill	2	3
Other or not disclosed	3	1
BV status at enrolment		
Normal	20	19
Intermediate	14	13
Positive	4	6
Missing information	0	1
Presence of HPV lesion at a study visit	0	0
Cervicitis at baseline	3	2
Cervicitis at last study visit	2	1
Regular partner		
Yes	30	32
No	4	5
Not disclosed	4	2
Number of sexual intercourse with regular partner during the last seven days (mean ± SD)	1.19 ± 1.1	1.26 ± 1.4
Number of participants who used condom with regular partner during the last seven days	5	5

ASA, acetylsalicylic acid; HCQ, hydroxychloroquine; HPV, human papilloma virus lesion was detected by villi/via test; n, number; SD, standard deviation; BV, bacterial vaginosis.

### 3.2 IA/inflammation

Systemic and mucosal T cell IA was assessed on fresh cells by flow cytometry, while inflammatory state was determined by measuring cytokine and chemokine proteins. The results presented herein compare IA after six weeks on drugs (visit 3) with baseline (visit 1).

### 3.3 HCQ arm

#### 3.3.1 Systemic HCQ analysis

At visit 3, 33/36 participants had detectable HCQ in the blood (mean 37.08 ng/mL) and 19/36 participants had detectable levels in CVL (mean 18,45 ng/mL). There were not enough biological samples to test three participants.

**Figure 2:**
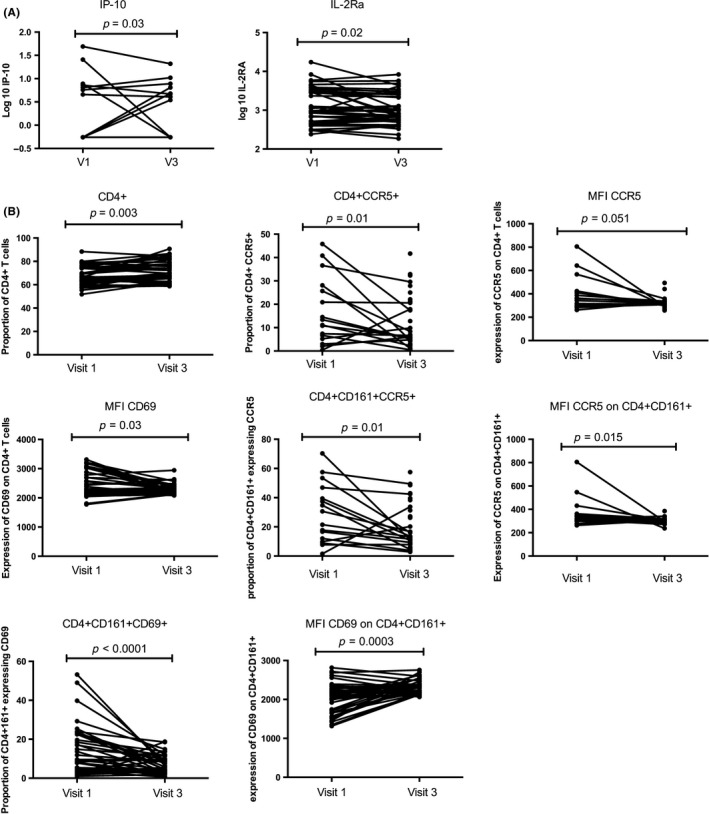
The impact of hydroxychloroquine (HCQ) treatment on the systemic immune system. **(A)** Systemic expression of pro‐inflammatory cytokines and chemokines. **(B)** CD4+T cells distribution and immune activation level at the systemic measured by flow cytometry. VI: represent baseline and V3 after six weeks on a daily HCQ (200 mg) treatment.

In the HCQ arm, the systemic level of two pro‐inflammatory chemokines IP‐10 (*p* = 0.03) and IL‐2Rα (*p* = 0.02) (Figure 2A) were significantly decreased following HCQ administration. We observed that there was a significant increase in the proportion of systemic T cells that were CD4+ (*p* = 0.003) but a significant reduction in the proportion of HIV target cells (defined as CD4+CCR5+CD3+) (*p* = 0.01) and Th17 (CD4+CD161+CCR5+) (*p* = 0.01) as well as lower proportion of activated Th17 cells (CD4+CD161+CD69+) (*p* < 0.0001). The intensity of expression of HIV co‐receptor CCR5 on a per cell basis, called mean fluorescence intensity (MFI), trended to be lower on CD4+ T cells (*p* = 0.051) and was significantly reduced on Th17 T‐cells (*p* = 0.015). Following six weeks of HCQ treatment, the intensity of expression of CD69 was significantly lower (*p* = 0.03 on CD4+ and *p* = 0.0003 on Th17) (Figure 2B). There was no significant correlation between the blood level of HCQ and the level of T cell IA markers.

As age and use of contraception are two factors that can influence IA, we performed multivariate linear regression to determine if the variations observed in the HCQ arm were influenced by either age or DMPA use. In our linear regression model, we observed that age influenced the proportion of CD4+ (*p *= 0.003), CD4+CCR5+ (0.01) and CD4+CD161+CCR5+ (*p* = 0.021) and the expression of CD69 on CD4+ T cells (*p *= 0.015) and CD4+CD161+ (0.001), while DMPA had an impact on the proportion of CD4+CD161+CD69+ T cells (*p* = 0.01) (data not shown). Only the expression of CCR5 on CD4+ T cells was not affected by age and DMPA as confounding factors. Due to the limitation of our study design and sample size, it is impossible to determine if the impact of age on the level of IA is stronger than the HCQ‐effect.

#### 3.3.2 Mucosal HCQ analysis

**Figure 3:**
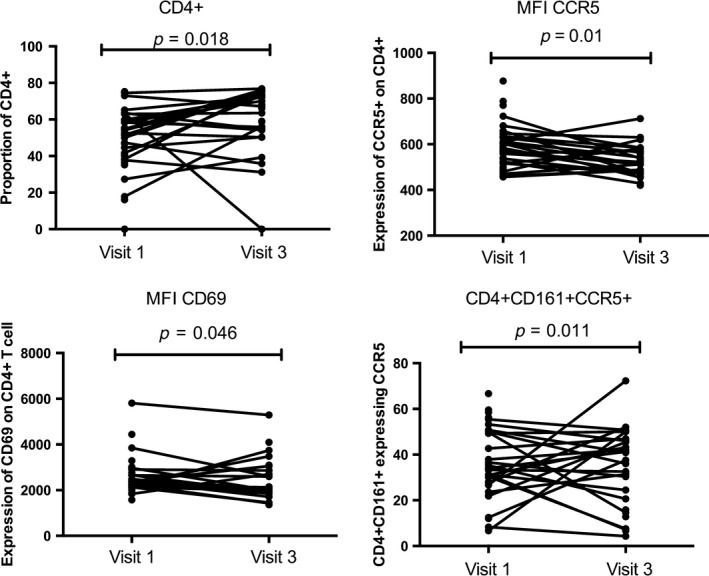
The impact of hydroxychloroquine (HCQ) on the immune activation at the female genital tract. Variation of CD4+T cells distribution at the female genital between baseline and six weeks on HCQ (200 mg) measured by flow cytometry, (visit 1 = baseline; visit 3: six weeks on HCQ treatment).

At the mucosal compartment, we did not observe any significant difference in the expression of cytokines/chemokines between baseline and visit 3. As observed in the blood, the proportion of T cells that were CD4+ was higher at visit 3 compared with baseline (*p* = 0.018). The proportion of Th17 cells expressing CCR5 (CD4+CD161+CCR5+) was significantly decreased at visit 3 (*p* = 0.011). The intensity of expression of the HIV co‐receptor CCR5 and the early activation/tissue residence marker CD69 were also reduced at visit 3 (*p* = 0.01 and *p* = 0.046 respectively) (Figure 3).

In the multivariate analysis, age affected the proportion of CD4+ (*p* = 0.008) and CD4+CD161+CCR5+ (*p* = 0.002). Neither age nor contraception affected the expression of CD69.

#### 3.3.3 Proteomics/microbiome in HCQ arm

A total of 401 unique human proteins were consistently identified in all CVL included in this study. Seventeen (4.2%) proteins were differentially abundant after six weeks of HCQ treatment compared with baseline levels (*p* < 0.05, paired t‐test, Table S1). Based on the distribution of *p*‐values from this data, we are unable to discern whether or not this study observed a true effect or if the results are due to random chance (Figure S1A,B). Proteins altered included increased levels of epithelial barrier proteins (TGM3, PPL, KRT1, CRNN, ACTN4) and decreased levels of immune response proteins (IGHG1, ELANE, ORM2, PGLYRP1). Ingenuity pathway analysis demonstrated that cell death (*p* = 0.0077, z = −2.04), the inflammatory response (*p* = 0.00913, z = −1.154), cell movement of leukocytes (*p* = 0.009, z = −1.12) and necrosis (*p* = 0.023, z = −1.02) functions were all found at lower levels post‐HCQ treatment (Table S2).

**Figure 4:**
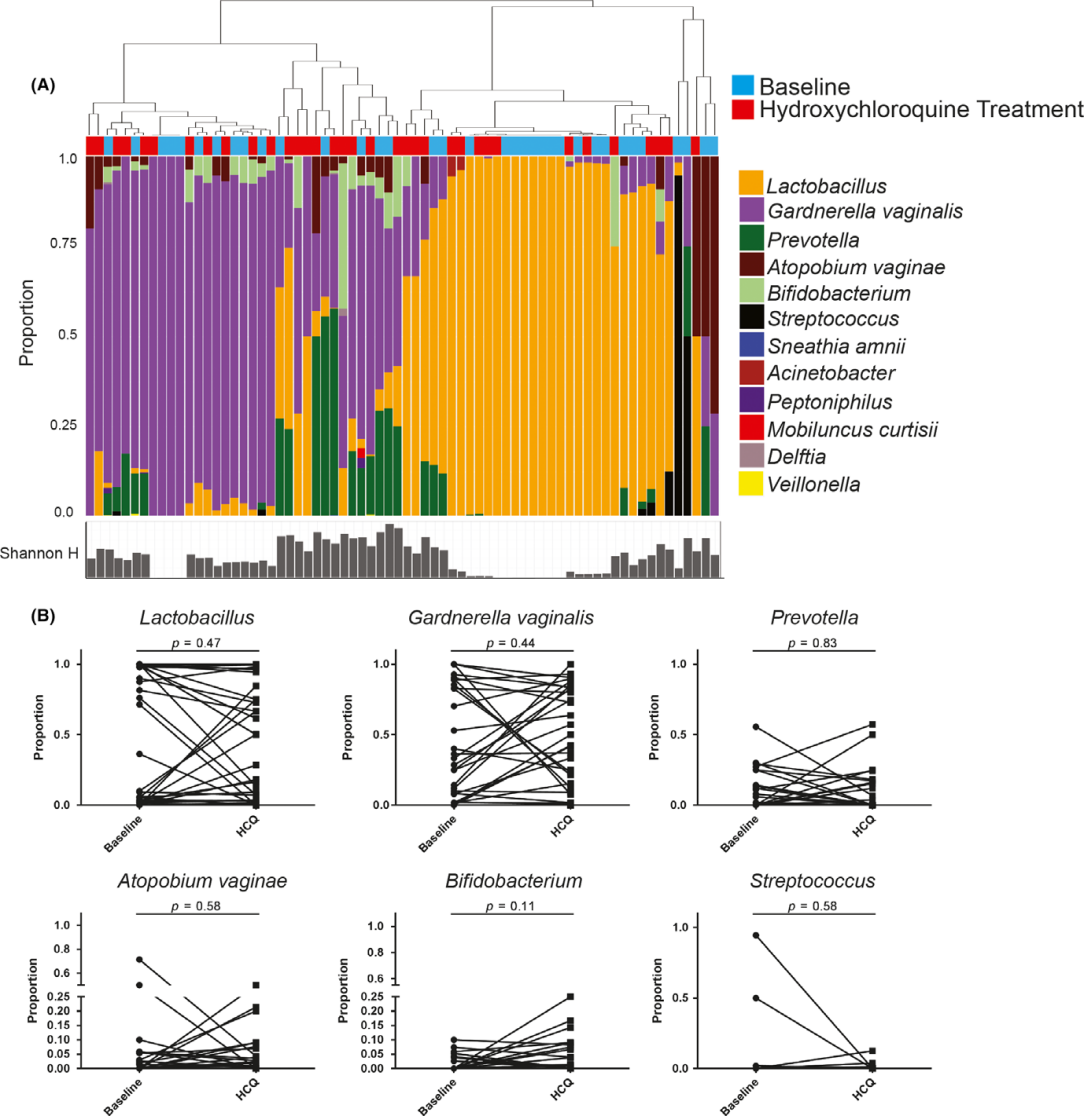
Vaginal bacterial profiles of women in the hydroxychloroquine (HCQ) arm. **(A)** Hierarchical clustering of microbial bacteria protein data from cervicovaginal secretions of women in the HCQ arm. Baseline samples are marked in blue and post‐treatment samples in red. Shannon's H index is indicated below each painter's plot. The distance metric utilized was Euclidean distance with average linkage. **(B)** Paired analysis of specific bacterial proportions from baseline and post‐treatment samples (Wilcoxon matched‐pairs signed‐rank test).

Mass spectrometry analysis identified 341 unique bacterial proteins belonging to 12 unique genera, and bacterial proportions are shown in Figure 4A. Samples belonged to two major groups, with bacterial communities dominated by *Lactobacillus* (n = 29, 42%) or non‐*Lactobacillus* taxa (n = 40, 58%) (>50% by proportion). Women with non*‐Lactobacillus* communities contained many bacterial vaginosis‐associated bacteria, which was predominantly *Gardnerella vaginalis*, but also included *Prevotella* and *Atopobium vaginae*. In the HCQ arm, the majority of women, 26 (74.2%), maintained a consistent microbial community type over the study, while four women (11.4%) changed from non‐*Lactobacillus*‐dominant (non‐LD) to *Lactobacillus*‐dominant (LD) after treatment, and five women (14.3%) changed from LD to non‐LD. There was no significant change to the proportion of specific bacteria taxa post‐HCQ treatment (Figure 4B).

### 3.4 ASA arm

#### 3.4.1 Systemic ASA analysis

ASA was detected in the plasma of 33/35 participants (mean 111.3 ng/mL) and in the CVL of 28/35 women (mean 46.14 ng/mL). The two participants with undetectable plasma levels had also undetectable CVL, likely indicating non‐compliance with taking the drug. We were unable to assess ASA concentration for three participants due to a lack of sample material.

**Figure 5:**
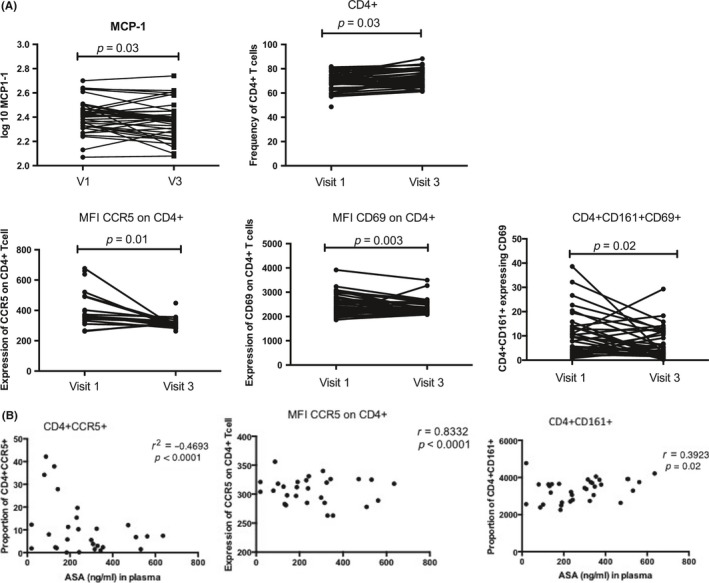
The impact of acetylsalicylic acid (ASA) on the systemic immune environment. **(A)** CD4+Tcells immune activation and level of cytokines/chemokines observed between visit 1 (baseline) and visit 3 (after six weeks on daily ASA treatment). **(B)** Spearman correlations between systemic drug level and systemic CD4+T cells activation.

In the plasma, the level of monocyte chemoattractant protein‐1 (MCP‐1/CCL2) was significantly decreased following six weeks on ASA (*p* = 0.03). There was an increased proportion of T cells expressing CD4+ compared with baseline (*p* = 0.03) and a significant decrease of Th17 cells (CD4+CD161+) that were of an activated phenotype (CD69+) (*p* = 0.02). Expression of CCR5 and CD69 on CD4+Tcells was also significantly lower compared with baseline (*p* = 0.01 and *p* = 0.003 respectively) (Figure 5A).

A multivariate regression analysis showed that neither age nor contraception impacted any of the changes observed between baseline and visit 3, suggesting the changes were likely directly due to ASA use.

The blood level of ASA concentration was negatively correlated with the proportion of HIV target cells (CD4+CCR5+) (*p* < 0.0001) but positively correlated with the intensity of expression of CCR5 on CD4+ T cells (*p* < 0.0001) and proportion of Th17 cells (*p* = 0.02) (Figure 5B).

#### 3.4.2 Mucosal ASA analysis

**Figure 6:**
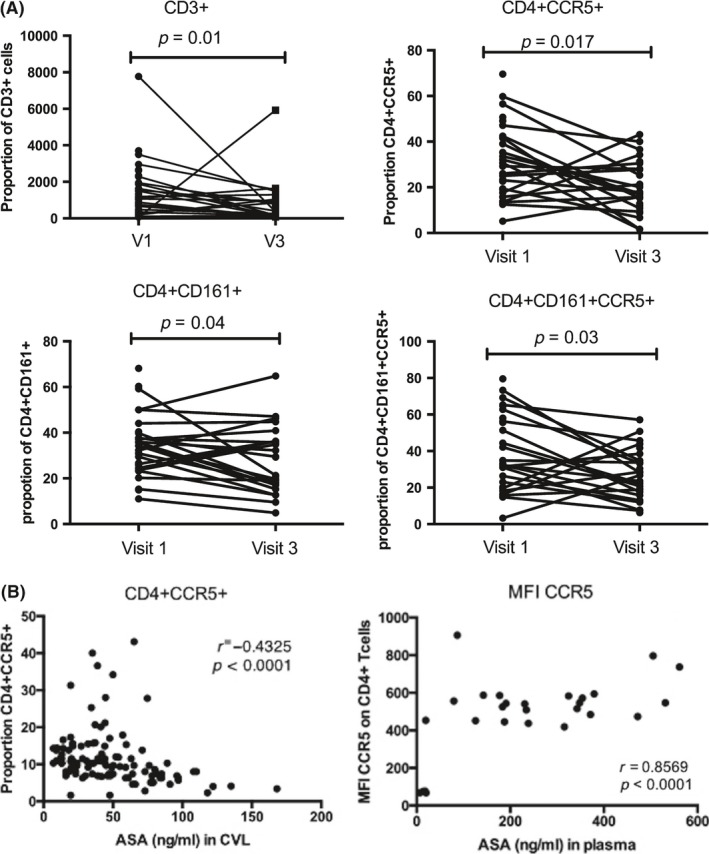
Impact of acetylsalicylic acid (ASA) on the mucosal compartment. **(A)** Level of immune activation at the female genital tract. Baseline (VI) compared with after six weeks of ASA treatment (V3). **(B)** Correlation of ASA concentration in the cervicovaginal lavage or plasma *versus* T cell (CD4+ and CCR5+) distribution observed at the female genital tract.

At the mucosal compartment following six weeks of ASA therapy, we observed significant reductions in the proportions of CD3+T cells (*p* = 0.01), Th17 (*p* = 0.04) and HIV target cells (CD4+CCR5+, *p* = 0.017; and CD4+CD161+CCR5+, *p* = 0.03) relative to baseline (Figure 6A).

Multivariate linear regression modelling showed that neither age nor contraceptive use affected the changes observed suggesting the effects were ASA‐associated.

We performed a correlation analysis to determine if the concentration of ASA had an impact on the IA observed at the female genital tract (FGT). The only marker that correlated with the systemic level of ASA was the expression of CCR5 on CD4+ Tcells. We also observed that the mucosal ASA concentration was inversely correlated with the proportion of HIV target cells at the FGT (*p* < 0.0001) demonstrating that higher mucosal level of ASA resulted in fewer HIV target cells at the genital tract (Figure 6B).

#### 3.4.3 Levels of mucosal HIV target cells in ASA treatment compared to HIV‐exposed seronegative women

**Figure 7:**
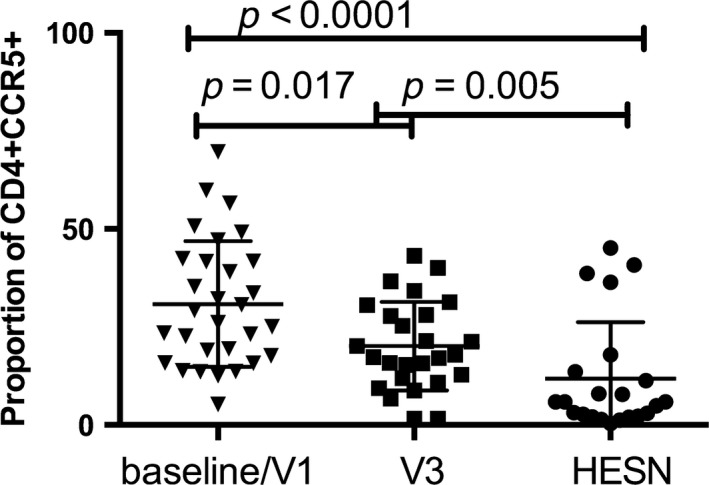
Proportion of HIV targets in HIV‐exposed seronegative (HESN). The proportion of CD4+ T cells that co‐express CCR5 at the female genital tract of non‐sex worker women in the acetylsalicylic acid (ASA) trial before (baseline/Vl) and six weeks (V3) following initiation of ASA and in HESN female sex workers from the Pumwani sex worker cohort.

Levels of mucosal CCR5+CD4+ T cells in low risk women from the general community in Nairobi declined significantly following six weeks of ASA therapy which itself was significantly higher than levels in HIV‐exposed seronegative (HESN) FSW in Nairobi using identical flow cytometry reagents, cytometer, and protocols (Figure 7).

#### 3.4.4 Proteomics/microbiome of ASA arm

**Figure 8:**
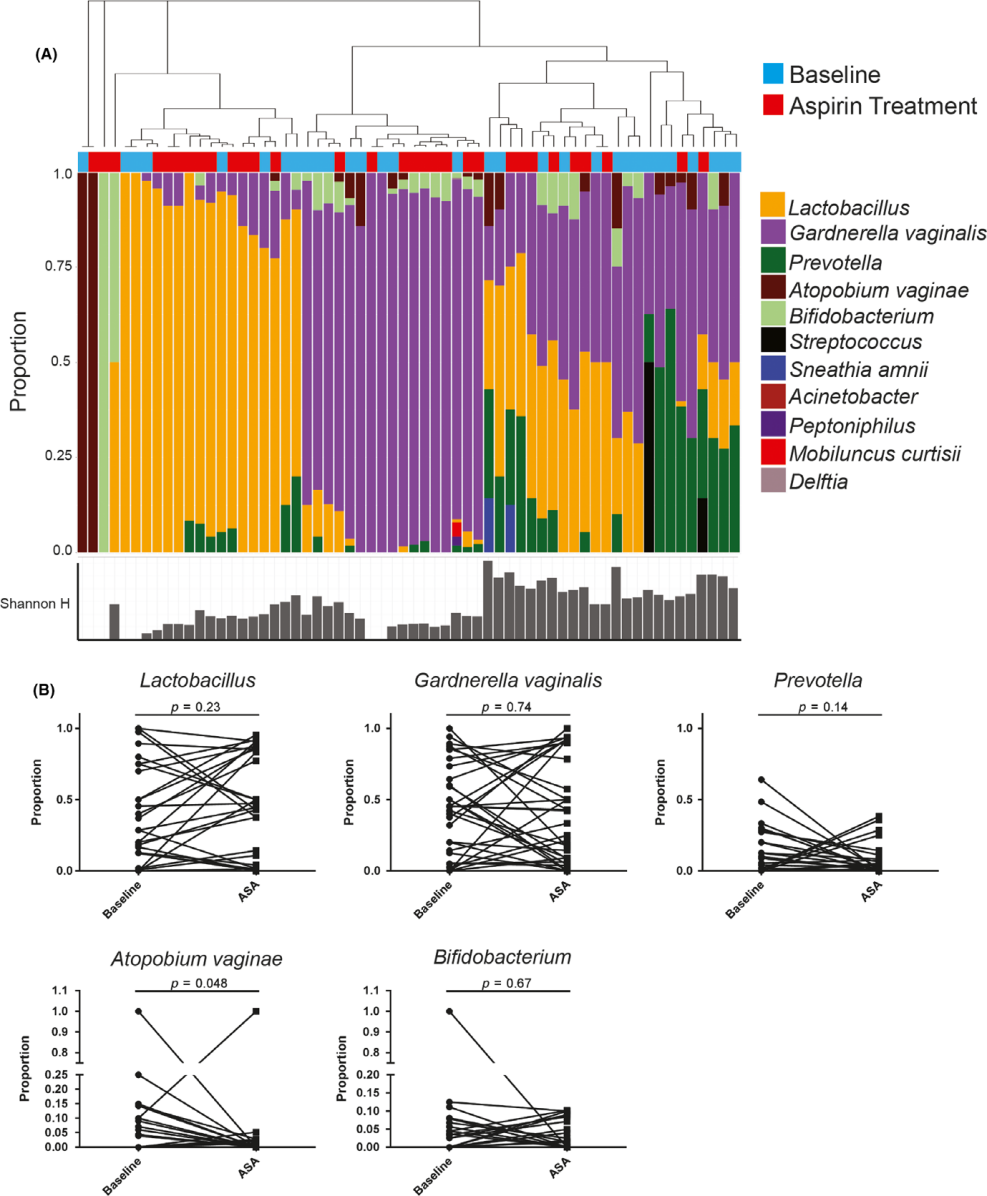
Vaginal bacterial profiles of women in the acetylsalicylic acid (ASA) arm. **(A)** Hierarchical clustering of microbial bacteria protein data from cervicovaginal secretions of women in the ASA arm. Baseline samples are marked in blue and post‐treatment samples in red. Shannon's H index is indicated below each painter's plot. The distance metric utilized was Euclidean distance with average linkage. **(B)** Paired analysis of specific bacterial proportions from baseline and post‐treatment samples (Wilcoxon matched‐pairs signed‐rank test).

The vaginal bacteria identified in CVL in the ASA arm are shown in Figure 8A, a similar pattern to the HCQ arm, where two major community groups were observed, including a minority that were *Lactobacillus*‐dominant (LD) (n = 22 or 33%) and the majority non‐*Lactobacillus* taxa dominant (non‐LD) (n = 45 or 67%). Non‐*Lactobacillus* communities contained high proportions of *Gardnerella vaginalis* and higher levels of *Prevotella* or *Atopobium vaginae* (Figure 8A). Of the women in the ASA arm, the majority (n = 24, 77.4%) maintained the same microbial community type over the study while four women (12.9%) changed from non‐LD to LD after treatment and three women (9.7%) changed from LD to non‐LD after treatment. Over the course of ASA use, there were no significant changes in the proportion of specific taxa with the exception of *Atopobium vagineae* (Figure 8B).

#### 3.4.5 Proteomics of host proteins in the ASA arm

**Figure 9:**
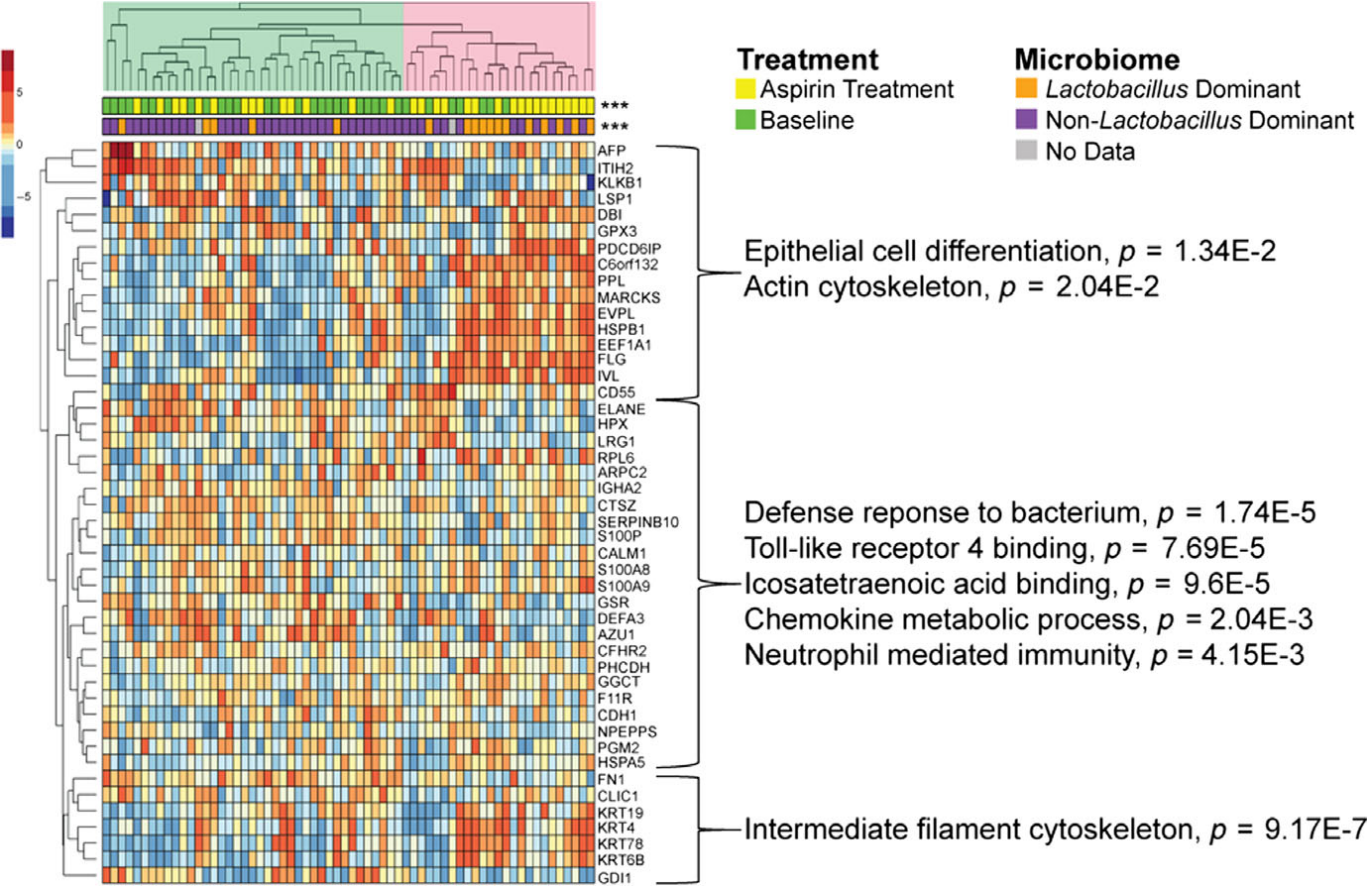
Vaginal proteome changes post‐acetylsahcyhc acid treatment. Heat map showing proteins differentially abundant (*p* < 0.05) post‐treatment in the acetylsalicylic acid arm were divided into two major branches by hierarchical clustering (depicted by green and pink trees). No proteins passed multiple comparison correction. Proteins in blue were significantly decreased post‐treatment and those in red were increased. Functional annotation of proteins found in major clusters were assigned using Consensus PathDB. Major microbiome profiles (*Lactobacillus* dominant or *non‐Lactobacillus* dominant) are indicated at the top of the heat map.

**Figure 10:**
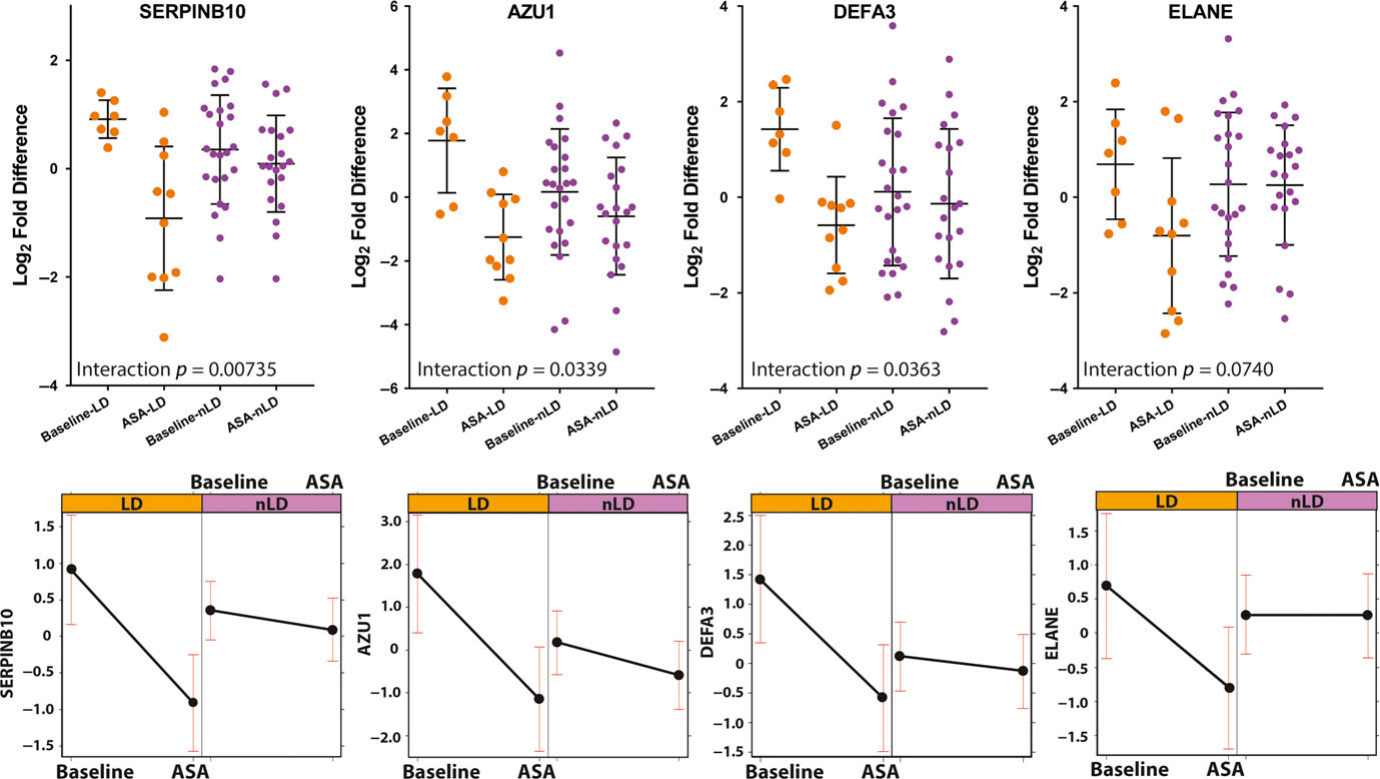
Interaction analysis showing how vaginal microbiome profiles modulate the anti‐inflammatory effects of ASA treatment. ASA, acetylsalicylic acid; LD, *Lactobacillus* dominant; Non‐LD, non‐*Lactobacillus* dominant.

Proteomic analysis of the ASA arm showed that after six weeks of ASA use, 46 (11.4%) human proteins were differentially abundant compared with baseline (*p* < 0.05, paired t‐test, Table S3, Figure 8A). A true effect was likely observed in this comparison as the distribution of *p*‐values below 0.05 are more than would be expected by random chance (Figure S1C,D). Hierarchical clustering showed these proteins separated baseline from post‐ASA treatment (visit 3) into two major branches (Figure 9). Proteins clustering with ASA treatment in branch 2 included those involved in cornification, epithelial barrier proteins (FLG, KRT6B, KRT78, KRT4, IVL, EVPL, PPL, KRT19), as well as decreased levels of inflammatory response (AZU1, KLKB1, DEFA3) and neutrophil degranulation (AZU1, LRG1, SERPINB10, ELANE, S100P) proteins. IPA analysis showed top pathways reduced post‐ASA treatment included recruitment of cells (*p* = 3.1E‐3, z = −2.2) and activation of phagocytes (*p* = 4.9E‐4, z = −2.17), while the concentration of lipid (*p* = 2.67E‐3, z = 2.16) was activated (Table S4). A higher proportion of LD women were observed in branch 2 (50%) compared with branch 1 (13%) (*p* = 2.9E‐3), where the highest levels of epithelial barrier proteins barrier proteins and reduced inflammation pathways were observed. In contrast, the majority of non‐LD samples (87%) were in branch 1 compared to branch 2 (50%). An interaction analysis of the 46 protein factors altered with ASA use, showed that five proteins had a significant interaction between treatment and microbiome profile (LD or non‐LD) (*p* < 0.05), with three additional factors trending at *p* < 0.1 (Table S3). These included soluble antimicrobial and immune cell recruitment factors SERPINB10, AZU1, ELANE and DEFA3 (Figure 10). The reduced expression of these factors with ASA treatment could largely be attributed to LD women, whereas treatment in non‐LD women appeared to have no effect on protein expression. A true effect was likely observed in this comparison as the distribution of *p*‐values below 0.05 are more than would be expected by random chance (Figure S1C,D). However, no proteins passed multiple comparisons correction.

## 4 Discussion

It has been well established that heterosexual HIV transmission is actually an infrequent event on a per sex act basis 14. The basic elements of transmission are a replication‐competent virus and a susceptible host cell. Most of the current biomedical prevention approaches focus on either keeping HIV away (condom use) or neutralizing it once it is present in the genital mucosa (PrEP, microbicides, vaccines). However, trying to limit the availability of susceptible HIV‐target cells at the genital tract, as a means of reducing HIV risk, has not been attempted. It is known that inflammation brings activated T cells to the site of irritation. Activated CD4+ T lymphocytes are more susceptible to HIV infection than quiescent cells and, once infected, produce up to 1000 times more virus 15. In this study, we show that taking low‐dose HCQ for six weeks was associated with a lower systemic IA, while low‐dose ASA consumption was associated with decreased systemic and mucosal HIV target cells.

ASA (81 mg) and HCQ (200 mg) were selected as they have both been used for long‐term prevention of chronic conditions with excellent safety records and were deemed acceptable and non‐stigmatizing in community consultations. Long‐term oral consumption of HCQ is clinically administered to control inflammation in autoimmune disease such as rheumatoid arthritis 16. Low‐dose ASA, commonly used to decrease risk of heart disease 17, 18 and it decreases inflammation through decreasing the production of prostaglandins and thromboxane A2 19, however, its action on T cell IA is unknown.

Due to the strong community engagement, for those that completed the study we observed an overall adherence rate of 93% (92% for the HCQ arm and 94% for the ASA) as determined by blood levels of drug. A novel observation was that both drugs were detected in the genital tract, with ASA having the better mucosal distribution.

Our findings indicate that a six‐week treatment of HCQ is associated with increased percentages of circulating CD4+ T cells, but overall those cells had lower levels of markers of IA (Figure 2). This supports a study from Piconi et al., who showed that higher dose of daily administered HCQ (400 mg) in HIV‐infected individuals had a notable reduction on IA and led to higher levels of regulatory T cells and lower T cell and monocyte activation 20. In our study among those taking HCQ, there was a 31% decrease in HIV target cells in the blood. At the genital tract, there was a 11% decrease in the density of the HIV co‐receptor CCR5 on CD4+ T cells, although the proportion of HIV target cells did not significantly decrease (Figure 3). The multivariate regression analysis performed for the HCQ arm showed the systemic and mucosal immune variations were confounded by age. The differences observed are most likely due to drug consumption, however, because of small sample size and study design we cannot exclude that different age groups might be affected differently by the study drug. HCQ treatment had no effect on the make‐up of the microbiome.

In participants who received ASA for six weeks, we observed decreased IA in the blood and mucosa. At the systemic environment, we observed lower levels of MCP‐1. MCP‐1 is a chemokine associated with inflammation and plays an important role in trafficking of monocytes and memory T cells 21. At the cellular level, we observed a lower proportion of activated Th17 cells in the blood after ASA treatment (Figure 5A). Interestingly, the intensity of expression of CCR5 was also decreased and this was negatively correlated with the concentration of ASA in the blood. At the genital tract, ASA consumption was associated with a decrease in the proportion of HIV target cells by 35% and Th17 cells by 28% (Figure 6A). It is not known if this decline would be sufficient to have an impact on HIV susceptibility, however, it does approach the level of HIV target cells found in HESN sex workers from Nairobi who display the immune quiescent phenotype (Figure 7). Whether a longer duration of ASA treatment would reduce the numbers of HIV target cells even further is not known. We detected ASA in the CVL of most participants (80%). Mucosal ASA concentration negatively correlated with the proportion of CCR5+CD4+ HIV target cells (Figure 6B). Interestingly, using a comprehensive systems biology assessment of human and bacterial proteins, we showed that although ASA use was not associated with a change in the microbiome, it was associated with a reduction in the quantity of host mucosal proteins involved in the inflammatory response and increased barriers function proteins, particularly in women with a *Lactobacillus*‐dominant vaginal microbiome (Figure 10). This suggests that ASA use may be associated with protection against HIV by decreasing inflammation as well as tightening the junction between epithelial cells making free virion migration into tissue more difficult. It also suggests that the microbiome may be an important determinant in pharmacological approaches to controlling inflammation.

### 4.1 Limitations

As with any study, we acknowledge the limitations of this study.

Although we did detect and control for many STIs and vaginal infections (HIV, candida, BV, trichomonas vaginalis, gonorrhoea, chlamydia and syphilis), as we did not measure active herpes simplex virus infection status in this cohort, we were not able to control for its potential confounding effects.

As the proteomic aspect of this study was intended to be a hypothesis generating discovery study, we evaluated hundreds of proteins. Therefore, when we corrected for multiple comparisons, none of the identified proteins remained significant. Any correlations observed must be confirmed in focused follow‐up studies.

It should be noted that not all individuals responded concordantly with the majority of those in the study. Although our study is under‐powered to directly address this issue, one could speculate that these discordant findings may be because some individuals experienced unreported infections, or were under stress, or fatigued, or had other environmental factors that affected their immune responses or their microbiome. We know that some of the participants did not take the drug as it was not detected in their blood, therefore their responses are likely to be discordant. It is also possible that for a subset of individuals their genetic background results in a decreased response to the drug. Larger follow‐up studies that have sufficient power to accommodate intra‐participant variation and that incorporate whole genome analysis should be designed to determine if there is a subset of people for whom this reducing inflammation/IIQ approach would not work.

Our study design did not include a placebo control arm. While this did limit the analyses that were available, we felt that as women vary in their baseline IA level, comparing each woman to herself before drug was biologically the best comparison and this was our first priority. Future studies should be higher powered and include a placebo‐control arm.

## 5 Conclusions

In conclusion, we showed that taking low‐dose HCQ and ASA was associated with a significant decrease in the proportion of HIV target cells in the blood and genital tract, respectively. We showed that ASA treatment was associated with an increase in the expression of protein involved in epithelial barrier while not altering the bacterial community. More studies are needed to understand the durability of the effects on target cell levels, determine optimal dosage and assess whether a similar impact on HIV target cell distribution can be observed in high‐risk FSW before moving on to an efficacy trial assessing HIV incidence. If reducing inflammation is proven to reduce HIV risk, it would be a new option for HIV prevention. There is no one‐size‐fits‐all approach to HIV prevention and it is clear that IIQ would not be the first method of prevention for everyone. While PrEP has tremendous potential for preventing HIV infections, it must be accepted by the community. In the sex worker cohort in Nairobi, we have seen that only 7% of high‐risk women offered PrEP were still taking it 12 months later. Work needs to be done to increase the acceptance of PrEP but clearly, other options that are acceptable to the community are needed. Our work with community suggests that anti‐inflammatory drugs would be highly acceptable. There may also be beneficial effects of using PrEP along with an anti‐inflammatory drug. A recent study from McKinnon et al. showed that genital inflammation among African women using anti‐HIV drug tenofovir in a vaginal gel reduced the effectiveness of the HIV prevention approach from 57% to 3% 22. If vaginal inflammation can be reduced it may actually aid other prevention approaches. At the recent International AIDS Society 2017 meeting in Paris, there was recognition that HIV prevention efforts, much like therapy, should include a cocktail of approaches, tailored to each person. For some individuals, using non‐stigmatizing anti‐inflammatory drugs, in addition to other HIV prevention approaches, may be an important addition to their HIV prevention tool kit.

## Competing interests

The authors have no conflicts of interest to declare.

## Authors’ contributions

JL, MK, JK and KR contributed to the study design. JL performed the data analysis, interpretation of the results and manuscript drafting. LM and GBL performed the experiments on cellular phenotyping. YC and EH performed the measurements of drugs. JC, MA, JM were involved in the recruitment and follow‐up of the participants. MK was the Medical officer of the study in charge of following participants. JO was the laboratory manager of the Nairobi laboratory. KB, LR, SM and AB performed proteomic and microbiome analysis, data interpretation and helped with manuscript writing. JK was the clinical leader, KF was the principal investigator of the study.

## Supporting information


**Figure S1.** Proteome changes in cervicovaginal lavage fluid of women in the HCQ and ASA treatment arms.

**Table S1.** Protein differently expressed at the genital tract after hydroxychloroquine treatment.
**Table S2.** Pathway affected by hydroxychloroquine treatment.
**Table S3.** Protein differently expressed at the genital tract after acetylsalicylic acid treatment.
**Table S4.** Pathway affected by acetylsalicylic acid treatment.

**Appendix S1.** Methods.

## References

[1] Unaids . Global aids up date 2016. Geneva: UNAIDS; 2016.

[2] Baral S , Beyrer C , Muessig K , Poteat T , Wirtz AL , Decker MR , et al. Burden of HIV among female sex workers in low‐income and middle‐income countries: a systematic review and meta‐analysis. Lancet Infect Dis. 2013;12(7):538–49. 10.1016/S1473-3099(12)70066-X 22424777

[3] Masson L , Mlisana K , Little F , Werner L , Mkhize NN , Ronacher K , et al. Defining genital tract cytokine signatures of sexually transmitted infections and bacterial vaginosis in women at high risk of HIV infection: a cross‐sectional study. Sex Transm Infect. 2014;90(8):580–7. 25107710 10.1136/sextrans-2014-051601

[4] Gosmann C , Anahtar MN , Handley SA , Farcasanu M , Abu‐Ali G , Bowman BA , et al. Lactobacillus‐deficient cervicovaginal bacterial communities are associated with increased HIV acquisition in young South African women. Immunity. 2017;46(1):29–37. 28087240 10.1016/j.immuni.2016.12.013 PMC5270628

[5] Zevin AS , Xie IY , Birse K , Arnold K , Romas L , Westmacott G , et al. Microbiome composition and function drives wound‐healing impairment in the female genital tract. PLoS Pathog. 2016;12(9):e1005889. 27656899 10.1371/journal.ppat.1005889 PMC5033340

[6] Naranbhai V , Abdool Karim SS , Altfeld M , Samsunder N , Durgiah R , Sibeko S , et al. Innate immune activation enhances hiv acquisition in women, diminishing the effectiveness of tenofovir microbicide gel. J Infect Dis. 2012;206(7):993–1001. 22829639 10.1093/infdis/jis465 PMC3501691

[7] Klatt NR , Cheu R , Birse K , Zevin AS , Perner M , Noël‐Romas L , et al. Vaginal bacteria modify HIV tenofovir microbicide efficacy in African women. Science. 2017;356(6341):938–45. 28572388 10.1126/science.aai9383

[8] McLaren PJ , Ball TB , Wachihi C , Jaoko W , Kelvin DJ , Danesh A , et al. HIV‐exposed seronegative commercial sex workers show a quiescent phenotype in the CD4+ T cell compartment and reduced expression of HIV‐dependent host factors. J Infect Dis. 2010;202 Suppl 3:S339–44. 20887221 10.1086/655968

[9] Card CM , McLaren PJ , Wachihi C , Kimani J , Plummer FA , Fowke KR . Decreased immune activation in resistance to HIV‐1 infection is associated with an elevated frequency of CD4(+)CD25(+)FOXP3(+) regulatory T cells. J Infect Dis. 2009;199(9):1318–22. 19301980 10.1086/597801

[10] Lajoie J , Juno J , Burgener A , Rahman S , Mogk K , Wachihi C , et al. A distinct cytokine and chemokine profile at the genital mucosa is associated with HIV‐1 protection among HIV‐exposed seronegative commercial sex workers. Mucosal Immunol. 2012;5(3):277–87. 22318497 10.1038/mi.2012.7

[11] Card CM , Ball TB , Fowke KR . Immune quiescence: a model of protection against HIV infection. Retrovirology. 2013;10(1):141. 24257114 10.1186/1742-4690-10-141 PMC3874678

[12] Juno JA , Boily‐Larouche G , Lajoie J , Fowke KR . Collection, isolation, and flow cytometric analysis of human endocervical samples. J Vis Exp. 2014;89:e51906–6. 10.3791/51906 PMC4212647 25045942

[13] Birse KD , Romas LM , Guthrie BL , Nilsson P , Bosire R , Kiarie J , et al. Genital injury signatures and microbiome alterations associated with depot medroxyprogesterone acetate usage and intravaginal drying practices. J Infect Dis. 2016;215:590–8. 10.1093/infdis/jiw590 PMC5388302 28011908

[14] Patel P , Borkowf CB , Brooks JT , Lasry A , Lansky A , Mermin J . Estimating per‐act HIV transmission risk: a systematic review. AIDS. 2014;28(10):1509–19. 24809629 10.1097/QAD.0000000000000298 PMC6195215

[15] Pan X , Baldauf H‐M , Keppler OT , Fackler OT . Restrictions to HIV‐1 replication in resting CD4+ T lymphocytes. Cell Res. 2013;23(7):876–85. 23732522 10.1038/cr.2013.74 PMC3698640

[16] Rainsford KD , Parke AL , Clifford‐Rashotte M , Kean WF . Therapy and pharmacological properties of hydroxychloroquine and chloroquine in treatment of systemic lupus erythematosus, rheumatoid arthritis and related diseases. Inflammopharmacology. 2015;23(5):231–69. 26246395 10.1007/s10787-015-0239-y

[17] Tchwenko S , Fleming E , Perry GS . Aspirin use for the primary prevention of myocardial infarction among men in North Carolina, 2013. Prev Chronic Dis. 2015;12:E202. 26583574 10.5888/pcd12.150342 PMC4655480

[18] Berkowitz AL , Westover MB , Bianchi MT , Chou SH‐Y . Aspirin for secondary prevention after stroke of unknown etiology in resource‐limited settings. Neurology. 2014;83(11):1004–11. 25122202 10.1212/WNL.0000000000000779 PMC4162302

[19] Vane JR , Botting RM . The mechanism of action of aspirin. Thromb Res. 2003;110(5–6):255–8. 14592543 10.1016/s0049-3848(03)00379-7

[20] Piconi S , Parisotto S , Rizzardini G , Passerini S , Terzi R , Argenteri B , et al. Hydroxychloroquine drastically reduces immune activation in HIV‐infected, antiretroviral therapy‐treated immunologic nonresponders. Blood. 2011;118(12):3263–72. 21576701 10.1182/blood-2011-01-329060

[21] Deshmane SL , Kremlev S , Amini S , Sawaya BE . Monocyte chemoattractant protein‐1 (MCP‐1): an overview. J Interferon Cytokine Res. 2009;29(6):313–26. 19441883 10.1089/jir.2008.0027 PMC2755091

[22] McKinnon LR , Liebenberg LJ , Yende‐Zuma N , Archary D , Ngcapu S , Sivro A , et al. Genital inflammation undermines the effectiveness of tenofovir gel in preventing HIV acquisition in women. Nat Med. 2018;24(4):491. 29480895 10.1038/nm.4506 PMC5893390

