# Supplementary figures and images for: Using safe, affordable and accessible non‐steroidal anti‐inflammatory drugs to reduce the number of HIV target cells in the blood and at the female genital tract
Source: J Int AIDS Soc. 2018 Jul 26;21(7):e25150. doi: 10.1002/jia2.25150 (PMC6060422; doi:10.1002/jia2.25150)

A

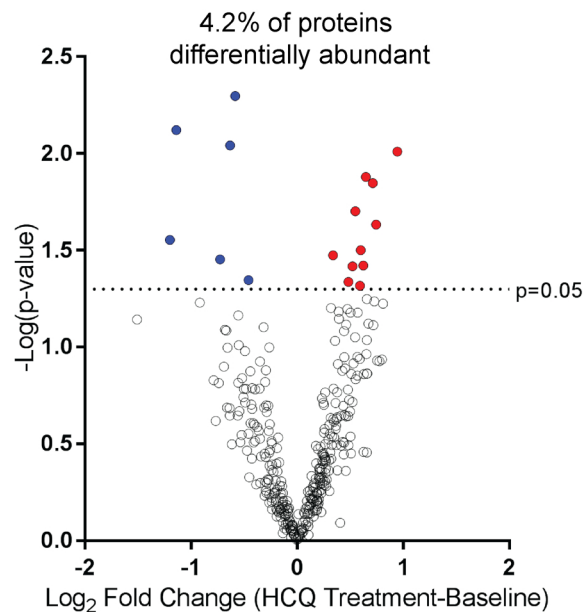

B

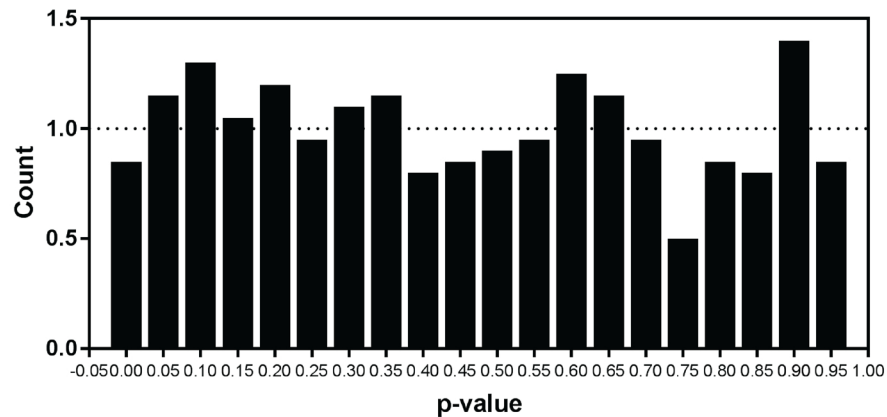

C

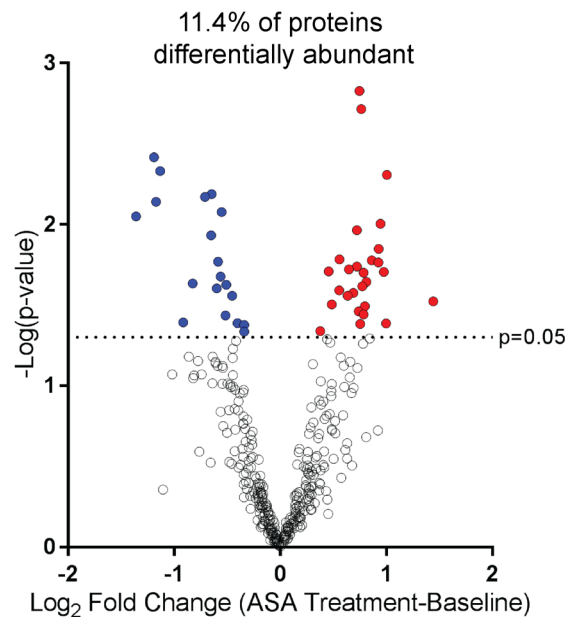

D

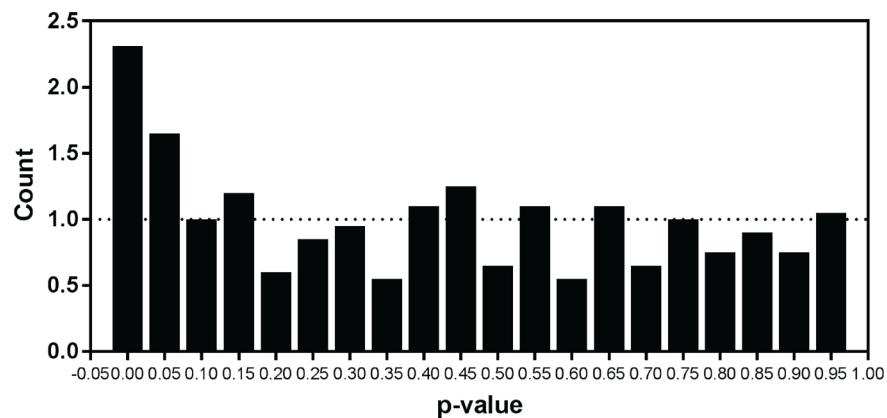

Supplement: Supplementary file 1 — Figure S1. Proteome changes in cervicovaginal lavage fluid of women in the HCQ and ASA treatment arms. [file JIA2-21-e25150-s001.pdf]
