# Supplementary material for: Using safe, affordable and accessible non‐steroidal anti‐inflammatory drugs to reduce the number of HIV target cells in the blood and at the female genital tract
Source: J Int AIDS Soc. 2018 Jul 26;21(7):e25150. doi: 10.1002/jia2.25150 (PMC6060422; doi:10.1002/jia2.25150)
Supplement: Supplementary file 2 — Table S1. Protein differently expressed at the genital tract after hydroxychloroquine treatment. Table S2. Pathway affected by hydroxychloroquine treatment. Table S3. Protein differently expressed at the genital tract after acetylsalicylic acid treatment. Table S4. Pathway affected by acetylsalicylic acid treatment. [file JIA2-21-e25150-s002.pdf]

Supplemental table 1: Protein differently expressed at the genital tract after HCQ treatment

Gene Name	Protein Name	FC Diff (HCQ-Baseline)	ttest pvalue (HCQ-Baseline)	ttest Sig	Interaction P value (HCQ*Microbiome)	Interaction Sig	General Function
PSMA7	Proteasome subunit alpha type-7	0.95	0.01	*	0.010	*	Host-virus interaction
RPL6	60S ribosomal protein L6	0.75	0.023	*	0.167		Translation
TGM3	Protein-glutamine gamma-glutamyltransferase E	0.71	0.014	*	0.941		Keratinization
PPL	Periplakin	0.65	0.013	*	0.418		Cornification
TUBA3E	Tubulin alpha-3E chain	0.63	0.038	*	0.188		Structural constituent of cytoskeleton
KRT1	Keratin, type II cytoskeletal 1	0.6	0.032	*	0.380		Establishment of skin barrier
CRNN	Cornulin	0.59	0.048	*	0.329		Cell-cell adhesion
NPEPPS	Puromycin-sensitive aminopeptidase	0.55	0.02	*	0.599		Cell growth
ACTN4	Alpha-actinin-4	0.52	0.038	*	0.770		Tight junction assembly
P4HB	Protein disulfide-isomerase	0.48	0.046	*	0.063	•	Protein folding
HSPA2	Heat shock-related 70 kDa protein 2	0.34	0.034	*	0.447		Stress response
AFM	Afamin	-0.46	0.045	*	0.035	*	Vitamin transport
IGHG1	Ig gamma-1 chain C region	-0.58	0.005	**	0.407		Immune response
ELANE	Neutrophil elastase	-0.63	0.009	**	0.424		ECM disassembly, positive regulation of immune response
ORM2	Alpha-1-acid glycoprotein 2	-0.73	0.035	*	0.278		Acute-phase response
PGLYRP1	Peptidoglycan recognition protein 1	-1.13	0.008	**	0.439		Immune response
SPRR3	Small proline-rich protein 3	-1.2	0.028	*	0.876		Keratinization

* interaction P<0.05

• interaction P<0.01

Supplemental table 2: Pathway affected by HCQ treatment

Biological Functions	p-Value	Activation z-score	Molecules	# Molecules
**cell death**	0.00773	**-2.044**	ACTN4,ELANE,HSPA2,IGHG1,P4HB,PGLYRP1,PSMA7,RPL6,TGM3	9
inflammatory response	0.00913	-1.154	ELANE,IGHG1,KRT1,PGLYRP1	4
cell movement of leukocytes	0.00904	-1.119	ACTN4,ELANE,IGHG1,PGLYRP1	4
necrosis	0.0229	-1.018	ELANE,IGHG1,P4HB,PGLYRP1,PSMA7,RPL6,TGM3	7

Legend: Blue: represent functions that are significantly inhibited and that pass the +/- 2 threshold for the z-score analysis from IPA

Supplemental table 3: Protein differently expressed at the genital tract after ASA treatment

Gene Name	Protein Name	FC Difference (ASA-Baseline)	ttest pvalue (ASA-Baseline)	Interaction P value (ASA*Microbiome)	Interaction Sig	General Function
LSP1	Lymphocyte-specific protein 1	1.44	0.0300	0.255		Neutrophil activation and chemotaxis
GPX3	Glutathione peroxidase 3	1.01	0.00494	0.833		Response to reactive oxygen species
FLG	Filaggrin	1.00	0.0410	0.542		Establishment of skin barrier
RPL6	60S ribosomal protein L6	0.98	0.0197	0.451		Translation
CD55	Complement decay-accelerating factor	0.94	0.00990	0.386		Complement activation
KRT6B	Keratin, type II cytoskeletal 6B	0.93	0.0142	0.749		Cornification
KRT78	Keratin, type II cytoskeletal 78	0.93	0.0172	0.681		Cornification
PDCD6IP	Programmed cell death 6-interacting protein	0.86	0.0167	0.00655	**	Bicellular tight junction assembly, apoptosis
KRT4	Keratin, type II cytoskeletal 4	0.81	0.0227	0.930		Cornification
ARPC2	Actin-related protein 2/3 complex subunit 2	0.80	0.0322	0.434		Actin filament polymerization
DBI	Acyl-CoA-binding protein	0.79	0.0199	0.0618	•	Transport
IVL	Involucrin	0.79	0.0361	0.606		Cornification
S100A9	Protein S100-A9	0.77	0.0241	0.790		Neutrophil chemotaxis, inflammatory response
CFHR2	Complement factor H-related protein 2	0.76	0.00193	0.595		Complement regulation
C6orf132	Uncharacterized protein C6orf132	0.75	0.0414	0.184		Unknown
EVPL	Envoplakin	0.75	0.00149	0.107		Cornification
EEF1A1	Elongation factor 1-alpha 1	0.74	0.0346	0.291		Protein biosynthesis
HSPB1	Heat shock protein beta-1	0.72	0.0109	0.248		Stress response
PPL	Periplakin	0.72	0.0183	0.479		Cornification
S100A8	Protein S100-A8	0.69	0.0266	0.716		Neutrophil chemotaxis, inflammatory response
MARCKS	Myristoylated alanine-rich C-kinase substrate	0.65	0.0190	0.704		Actin crosslink formation

KRT19	Keratin, type I cytoskeletal 19	0.64	0.0277	0.609		Cornification
PHGDH	D-3-phosphoglycerate dehydrogenase	0.56	0.0165	0.381		Amino acid biosynthesis
CTSZ	Cathepsin Z	0.56	0.0257	0.634		Proteolysis
CALM1	Calmodulin-1	0.49	0.0313	0.530		Negative regulation of ryanodine-sensitive calcium-release channel activity
IGHA2	Immunoglobulin heavy constant alpha 2	0.46	0.0196	0.0624	•	Immune response
GGCT	Gamma-glutamylcyclotransferase	0.38	0.0459	0.566		Glutathione biosynthetic process
HSPA5	78 kDa glucose-regulated protein	-0.34	0.0461	0.0871	•	Protein folding
S100P	Protein S100-P	-0.34	0.0420	0.120		Neutrophil degranulation
F11R	Junctional adhesion molecule A	-0.40	0.0409	0.373		Bicellular tight junction assembly
ELANE	Neutrophil elastase	-0.45	0.0276	0.0740	•	Neutrophil degranulation
PGM2	Phosphoglucomutase-2	-0.51	0.0236	0.0479	*	Glucose metabolism
CLIC1	Chloride intracellular channel protein 1	-0.52	0.0367	0.953		Ion transport
CDH1	Cadherin-1	-0.55	0.00836	0.441		Cell adhesion
NPEPPS	Puromycin-sensitive aminopeptidase	-0.56	0.0210	0.0867	•	Proteolysis
HPX	Hemopexin	-0.58	0.0170	0.102		Positive regulation of interferon-gamma-mediated signaling pathway
DEFA3	Neutrophil defensin 3	-0.60	0.0249	0.0363	*	Innate immune response in mucosa
SERPINB10	Serpin B10	-0.65	0.00649	0.00735	**	Neutrophil degranulation
FN1	Fibronectin	-0.65	0.0117	0.797		ECM disassembly
LRG1	Leucine-rich alpha-2-glycoprotein	-0.71	0.00675	0.971		Neutrophil degranulation
GSR	Glutathione reductase, mitochondrial	-0.83	0.0232	0.447		Response to reactive oxygen species
GDI1	Rab GDP dissociation inhibitor alpha	-0.91	0.0405	0.754		Signal transduction
KLKB1	Plasma kallikrein	-1.13	0.00467	0.520		Inflammatory response
ITIH2	Inter-alpha-trypsin inhibitor heavy chain H2	-1.17	0.00722	0.190		Hyaluronan metabolism

AZU1	Azurocidin	-1.19	0.00383	0.0339	*	Inflammatory response
AFP	Alpha-fetoprotein	-1.36	0.00892	0.375		Transport

** interaction *P*<0.01
* interaction *P*<0.05
• interaction *P*<0.01

Supplemental table 4: Pathway affected by ASA treatment

Biological Functions	p-Value	Activation z-score	Molecules	# Molecules
**concentration of lipid**	0.00267	**2.161**	AFP,DBI,EEF1A1,KLKB1,PDCD6IP,PHGDH,S100A8,S100A9	8
replication of Retroviridae	0.000395	1.98	DEFA3 (includes others),PDCD6IP,S100A8,S100A9	4
replication of RNA virus	0.00277	1.741	DEFA3 (includes others),EEF1A1,F11R,PDCD6IP,S100A8,S100A9	6
replication of virus	0.000941	1.331	DEFA3 (includes others),EEF1A1,F11R,FLG,PDCD6IP,S100A8,S100A9	7
formation of filaments	0.00526	1.084	ARPC2,FN1,HSPA5,HSPB1,PDCD6IP	5
damage of epithelial tissue	6.20E-06	-1	AZU1,ELANE,F11R,HPX,KRT6B	5
chemotaxis	0.000153	-1.015	AFP,AZU1,DEFA3 (includes others),ELANE,FN1,LSP1,S100A8,S100A9	8
migration of endothelial cells	0.000278	-1.053	F11R,FN1,HSPA5,HSPB1,MARCKS,S100P	6
transmigration of cells	0.000787	-1.184	CDH1,ELANE,F11R,FN1	4
killing of cells	0.000252	-1.296	AZU1,CDH1,DEFA3 (includes others),ELANE,HSPA5	5
metabolism of reactive oxygen species	0.000524	-1.474	CLIC1,DBI,ELANE,FN1,GPX3,HSPB1,S100A8	7
synthesis of reactive oxygen species	0.00241	-1.474	CLIC1,DBI,ELANE,FN1,HSPB1,S100A8	6
cell survival	0.000903	-1.514	CD55,CDH1,DEFA3 (includes others),ELANE,FN1,GSR,HSPA5,HSPB1,KRT19,S100A8,S100A9,S100P	12
migration of cells	8.51E-06	-1.544	ARPC2,AZU1,CD55,CDH1,CTSZ,DEFA3 (includes others),ELANE,F11R,FN1,HSPA5,HSPB1,KRT19,KRT6B,LSP1,MARCKS,S100A8,S100A9,S100P	18
inflammatory response	0.000081	-1.559	AZU1,DEFA3 (includes others),ELANE,F11R,FN1,HSPB1,KLKB1,LSP1,S100A8,S100A9	10

cell movement of myeloid cells	3.50E-06	-1.612	AZU1,CD55,DEFA3 (includes others),ELANE,F11R,FN1,HSPB1,LSP1,S100A8,S100A9	10
activation of cells	0.00012	-1.616	AFP,AZU1,CD55,CDH1,CLIC1,ELANE,FN1,KLKB1,LSP1,S100A8,S100A9	11
cell movement	4.66E-07	-1.84	AFP,ARPC2,AZU1,CD55,CDH1,CTSZ,DEFA3 (includes others),ELANE,F11R,FN1,GDI1,HSPA5,HSPB1,KLKB1,KRT19,KRT6B,LSP1,MARCKS,S100A8,S100A9,S100P	21
cell viability	0.00186	-1.882	CD55,CDH1,DEFA3 (includes others),FN1,GSR,HSPA5,HSPB1,KRT19,S100A8,S100A9,S100P	11
activation of myeloid cells	0.00032	-1.944	AZU1,ELANE,FN1,KLKB1,S100A8,S100A9	6
aggregation of cells	0.000293	-1.953	CDH1,CLIC1,FN1,GPX3,HSPB1,KLKB1	6
activation of blood cells	0.00131	-1.953	AZU1,CD55,CLIC1,ELANE,FN1,KLKB1,S100A8,S100A9	8
activation of leukocytes	0.00382	-1.953	AZU1,CD55,ELANE,FN1,KLKB1,S100A8,S100A9	7
adhesion of epithelial cells	0.000163	-1.961	CDH1,ELANE,FN1,PDCD6IP	4
**activation of phagocytes**	0.000491	**-2.173**	AZU1,ELANE,FN1,KLKB1,S100A8,S100A9	6
**recruitment of cells**	0.00307	**-2.2**	ELANE,FN1,KLKB1,LSP1,S100A8	5

Legend:red represents activated and blue represents inhibited functions that pass the +/- 2 threshold for the z-score analysis from IPA.
