# Supplementary material for: Using safe, affordable and accessible non‐steroidal anti‐inflammatory drugs to reduce the number of HIV target cells in the blood and at the female genital tract
Source: J Int AIDS Soc. 2018 Jul 26;21(7):e25150. doi: 10.1002/jia2.25150 (PMC6060422; doi:10.1002/jia2.25150)
Supplement: Supplementary file 3 — Appendix S1. Methods. [file JIA2-21-e25150-s003.docx]

**Using safe, affordable and accessible non-steroidal anti-inflammatory drugs to reduce the number of HIV target cells in the blood and at the female genital tract.** J Lajoie et al

**Supplemental Methods:**

***Entry criteria:***

Entry criteria were age greater than 18 and less than 55, not self-declaring as a sex worker, presence of uterus and cervix, willingness to adhere to study protocol, in general good health, not currently taking anti-inflammatory or immunosuppressant drugs, being HIV uninfected and not having history of cardiovascular diseases. Exclusion criteria included being pregnant in the last 12 months, the presence of a sexual transmissible infection (STI) at enrolment or at any time during the course of the study, menopause, taking medication that counteract the study drugs, being allergic to the study drugs, having history of heartburn, stomach pain, stomach ulcer, anemia, haemophilia, kidney or liver disease, psoriasis, G-6-PD deficiency, dermatitis, alcoholism, cardiovascular diseases, eye disease or sight impairment or being currently involved in another clinical trial.

***Randomization methods:***

Eligible participants were entered and randomized in the study within two weeks, using randomization software (www.randomization.com).We linked participant ID with a study drug. Each participant’s ID was inscribed on a sealed envelope with the drug arm information. At enrolment, the clinical staff attributed the next available ID to the new participant and opened the enveloped to determine the study arm.

***Study procedures:***

Participants were randomized to receive either oral ASA (81mg) (Bayer Canada, Mississauga, Canada) or HCQ (200mg) (Sanofi Aventis, Paris, France) once a day for a period of six weeks. Participants were pre-screened for STIs including HIV. Eligible participants were randomized using randomization software (www.randomization.com). Participants were followed for a total of 12 weeks (4 pre-drug, 6 on drug, 2 post-drug) with sampling occurring monthly. Women not on DMPA were asked to come 5-10 days after the start of menstrual bleeding to ensure that all mucosal samples were taken during the follicular phase of the cycle. Women on DMPA were only enrolled if on therapy for at least two weeks. As they did not have a menstrual cycle, their samples were taken any time. Visit 1 was the pre-drug visit and Visit 3 was following 6 weeks on drug therapy. At Visit 1, we assessed systemic and mucosal baseline immune activation of each participant, and each participant served as her own control by comparing the pre-drug baseline values. To assess for adherence, a pill count was performed at each visit. Subsequently plasma and cervico-vaginal lavage (CVL) were shipped to Winnipeg, Canada for drug level measurements.

***Drug measurement:***

ASA HPLC analysis was performed using a Symmetry® C18 column (300Å, 3.5 μm 4.6 mm x

75 mm; Waters) with a Symmetry® C18 guard column (300Å, 5 μm, 3.9 mm x 20 mm; Waters), fitted to a Waters® Alliance® HPLC system equipped with Waters® 2690 Separations module and Waters® 996 Photodiode Array detector. The mobile phase for ASA consisted of water, acetonitrile and orthophosphoric acid at a ratio of 74:18:0.9 (v/v, pH 2.5, quantitated at 234 nm).

Each 100μL injection was run for 10 min at 1 mL/min. The retention time for the internal standard simvastatin (SIM) and ASA were 0.97 min and 5.09 min, respectively. The detection wavelength was set at 234 nm. For ASA plasma sample preparation, 200μL plasma was combined with equivalent volume of internal standard solution. pH of the entire solution was adjusted to 2.7 by the addition of orthophosphoric acid. Analyte was extracted using 400 μL of acetonitrile followed by mixing and centrifugation. The supernatant was then transferred into a microcentrifuge tube containing 100-120 mg of sodium chloride. After vortexing and centrifugation, 100 μL of the upper organic phase was injected for HPLC analysis. CVL samples were extracted similarly except the resulting supernatant was collected and evaporated under nitrogen gas. Following the addition of 100 μL of 0.01 M hydrochloric acid into the vial for reconstitution, the entire solution was injected into the HPLC machine. For ASA, the lower limits of quantitation (LLOQ) in plasma and CVL are 78 ng/mL and 39 ng/mL with extraction efficiency of 86.45 ± 1.31 % (mean ± SD) and 92.16 ± 0.64 %, respectively.

HCQ was analyzed under a gradient elution using the same HPLC system and columns with internal standard chloroquine (CQ). The aqueous mobile phase consisted of 58 mM monobasic sodium phosphate buffer to which 15 mM heptanesulfonic acid sodium salt was added and adjusted to pH 3.1 with concentrated phosphoric acid (mobile phase A). Organic phase consisted of a mixture of acetonitrile and methanol (85:15, mobile phase B). The mobile phase was programmed to be delivered at a flow rate of 1 mL/min at the following gradient condition: 100% A at 0 min; 83:17 (A:B, the same for below) at 5 min; 75:25 at 17 min; 70:30 at 21 min; 65:35 at 30 min. Each 100μL injection was run for 30 min. UV detection was set at 343 nm. The retention time of CQ and HCQ was approximately 14 min and 18 min, respectively. The plasma sample preparation involved sequentially combining 500 μL plasma with 100μL internal standard solution, 4 mL of diethyl ether, and 1 mL of 0.25 M sodium hydroxide. After being vortexed and centrifuged, the organic phase was evaporated and reconstituted in 300μL of mobile phase. For human CVL samples, the resulting organic phase was completely evaporated and reconstituted with 100 μL of mobile phase. For HCQ, the LLOQ in plasma and CVL are 44 ng/mL and 24 ng/mL with extraction efficiency of 81.87 ± 2.10 % and 90.54 ± 0.95 %, respectively. Values below the specified LLOQ were reported as half the limit of detection.

***Drug level detection:***

Levels of ASA and HCQ in plasma and CVL samples were quantitated using reversed phase high-performance liquid chromatography (RP-HPLC) following previously described methods with modifications^13,14^. Drug contents in the samples were extracted and analyzed using the Symmetry^®^ C18 column attached to a Waters^®^ Alliance^®^ HPLC system equipped with Waters^®^ 2690 Separations module and Waters^®^ 996 Photodiode Array detector. Simvastatin and chloroquine were used as internal standards for ASA and HCQ, respectively (see supplemental methods for details).

***Participant characteristics:***

All women were screen for syphilis and none were positive. Twenty-seven participants were prematurely discontinued from the study for the following reasons: 7 withdrew consent (6.7%), 8 were discontinued due to positive STI diagnosis (7.6%) and 12 participants were lost to follow-up (11.5%).

Presence of cervical inflammation was assessed by a visual exam of the cervix. At baseline, 5 participants were positive for cervicitis and two of them still had cervical inflammation at the last cervical inspection (Visit 3). BV was assessed at each visit and Table 1 indicates the status at baseline. Between Visit 1 and Visit 3, the BV status of 26 participants changed and yet there was overall no significant difference in the variation of the BV status for either study arm over the course of the study. Self-reported drug adherence was similar between the two arms.

***Community engagement:***

From its inception phase, the present study was created with the input of Kenyan FSW and women from the general community to ensure the feasibility and acceptability of this new approach. As FSW are 13 times more likely to be HIV infected than the general population, the opinion of the FSW communities was important to get insights about the challenges they anticipated and the long-term feasibility of this strategy. We conducted this study among non-sex workers, as our previous work has shown they have elevated levels of immune activation relative to FSWs. After community consultations, the major feedback we received was the importance of trying to develop a new tool to prevent HIV that was not associated with stigma around HIV prevention such as ARVs. The ease of access, low cost, safety profile and lack of stigma associated with their use were all reasons the community provided for why ASA and HCQ would be suitable anti-inflammatory drugs for the study.

***Mass spectrometry analysis:***

Mass spectrometry was utilized to characterize both human and microbial proteins. Briefly, 100μg of protein from each sample were digested with trypsin and analyzed by tandem mass spectrometry using an Orbitrap Velos mass spectrometer. Human peptide identity searching was performed using Mascot v2.4.0 against the SwissProt database restricting taxonomy to Human. Bacterial peptide identity searches were performed using a manually curated TrEMBL database containing the major identified genera identified from an initial search (17 genera total, 4,206,764 proteins total). Search results were imported into Scaffold to validate the protein identifications, using the following criteria: ≤0.1% False Discovery Rate (FDR) for peptide identification, ≤1% FDR for protein identification, and at least 2 unique peptides identified per protein. Human proteome results were imported into Progenesis LC-Mass Spectrometry (MS) software to perform label-free differential protein expression analysis based on MS peak intensities. Feature detection, normalization, and quantification were all performed using default settings from the software. Microbial abundance was calculated by summing normalized total spectral counts from Scaffold for all proteins associated with each genus. Functional annotation of proteins was performed using over-representation analysis from ConsensusPathDB and Ingenuity Pathway Analysis.

Each fraction was separately analysed using a nano-flow Easy nLC II connected in-line to an

LTQ Orbitrap Velos mass spectrometer with a nanoelectrospray ion source at 2.35 kV (Thermo

Fisher Scientific, San Jose, CA, USA). The peptide fractions were loaded (1 μg) onto a C18-reversed phase trap column (3 cm long, 100 μm inner diameter, 5 μm particles) with 100% buffer A (2% acetonitrile, 0.1% formic acid) at 6μl/min for a total volume of 30 μl, and then separated on a C18-reversed phase column (15 cm long, 75 μm inner diameter, 2.4 μm particles).

Both columns were packed in-house with ReproSil-Pur C18-AQ resin (Dr. Maisch). Peptides were eluted using a linear gradient of 2-32% buffer B (98% acetonitrile, 0.1% formic acid) over 120 min at a constant flow rate of 250 nl/min. Total LC/MS/MS run-time was 160 minutes, including the loading, linear gradient, column wash at 95% buffer B, and the equilibration.

Data were acquired using a data-dependent method, dynamically choosing the top 10 abundant precursor ions from each survey scan for isolation in the LTQ Velos (2.0 m/z isolation width) and fragmentation by CID (35% normalized collision energy, with 10 ms activation time). The survey scans were acquired in the Orbitrap over m/z 300-1700 with a target resolution of 60000 at m/z 400, and the subsequent fragment ion scans were acquired in the LTQ Velos iontrap. The lower threshold for selecting a precursor ion for fragmentation was 1000 ions. Dynamic exclusion was enabled using a list size of 500 features, a m/z tolerance of 15 ppm, a repeat count of 1, a repeat duration of 30 s, and an exclusion duration of 15 s.

***MS Database Construction & Taxonomic (Bacteria) Search Details:***

Initial searches were performed using the Mascot search engine (v2.4, Matrix Science) against the TrEMBL database (September-2016) that was restricted to only include bacterial proteins both from reviewed and non-reviewed sources (Database construction parameters: taxonomy:bacteria AND reviewed:no, downloaded from Uniprot). Using these results, we generated a new, curated database (4,206,764 proteins) containing genera identified from our initial search. (Most abundant genus to least: Lactobacillus, Gardnerella, Acinteobacter, Prevotella, Streptococcus, Bifidobacterium, Mobiluncus, Nocardia, Bradyrhizobium, Sneathia, Alloscardovia, Petptoniphilus, Atopobium, Megasphaera, Comamonadaceae, and Veillonella).

Bacterial databases were also merged with the UniProtKB Human database to account for homologous proteins between the bacterial genera and humans. The "OR" function was also set as a database construction parameter to limit the inclusion of homologous proteins among the bacterial genera included. Individual samples were searched a second time against this curated database along with a decoy database to determine the number of false discoveries. Search results were imported into Scaffold (v 4.4.1, Proteome Software). Confidence thresholds were set to ≤1% FDR protein identification, requiring at least 2 unique peptides and ≤0.1% FDR peptide identification. Accession number protein reports were further monitored to remove any protein identifications that were assigned to more than one genus. Criteria for assigning presence of microbial proteins included those that had at least 1 peptide in its samples, and at least two peptides identified per protein across all samples. Microbial abundance was calculated by summing normalized total spectral counts for all proteins associated with each genus.

Relatedness between microbial groups was determined by unsupervised hierarchical linkage clustering using average Euclidean linkage as the distance metric. Graphical representations were generated in GraphPad Prism (version 6.05).

***Statistical analysis:***

Immune markers normally distributed:

| ***ASA*** | **Normally distributed** |
| --- | --- |
| *cytokines in the CVL* | IL-1a (log transformed) |
|  | IP-10 (log transformed) |
|  | MCP-1 (log transformed) |
|  |  |
| *cytokines in the plasma* | IP-10 (log transformed) |
|  | MCP-1 (log transformed) |
|  | MIG (log transformed) |
|  |  |
| *CMC* | CD4+ frequency |
|  | CD4+CCr5+ frequency |
|  | CD4+CD161+ frequency |
|  | CD4+CD161+CCR5+ frequency |
|  |  |
| *PBMC* | CD4+ frequency |
|  |  |
| ***HCQ*** |  |
|  |  |
| *Cytokines in the CVL* | IL-1a (log transformed) |
|  | IL-8 (log transformed) |
|  | IP-10 (log transformed) |
|  | MCP-1 (log transformed) |
|  |  |
| *Cytokines in the plasma* | IL-2Ra (log transformed) |
|  |  |
| *CMC* | CD4+CCR5+ frequency |
|  | CD4+CD69+ frequency |
|  | CD4+CD161+CCR5+ frequency |
|  |  |
| *PBMC* | CD4+ frequency |
|  |  |
| Legend: If not mentioned in this table, markers were not normally distributed | |
